# Light mowing supports fine-root production in a temperate saline-alkaline grassland soil

**DOI:** 10.3389/fpls.2026.1892574

**Published:** 2026-07-15

**Authors:** Meng Cui, Huajie Diao, Qingshi Ning, Yingzhi Gao

**Affiliations:** 1 College of Grassland Science, Xinjiang Agricultural University, Key Laboratory of Grassland Resources and Ecology of Xinjiang Uygur Autonomous Region, Urumqi, Xinjiang, China; 2 Coll Desert Control Sci & Engn, Inner Mongolia Agricultural University, Hohhot, China; 3 College of Grassland Science, Shanxi Agricultural University, Taigu, China; 4 State Key Laboratory of Urban and Regional Ecology, Research Center for Eco-Environmental Sciences, Chinese Academy of Sciences, Beijing, China

**Keywords:** mowing intensity, root production, root seasonal dynamics, root turnover, saline- alkaline grassland

## Abstract

The dynamic changes and differential responses of roots - an important research hotspot in belowground ecology-are one of the most fascinating yet elusive focuses in terrestrial ecology. We conducted a two-year field experiment in a temperate saline-alkaline grassland soil of northern China to generalize the effects of mowing intensity on root production, mortality, standing crop, turnover, and lifespan. Four mowing intensities were established, including no mowing, light mowing, moderate mowing, and heavy mowing. Fine root dynamics were monitored *in situ* using the root-window method at 15-day intervals from May 2018 to October 2019, and measurements were performed in the 0–10 cm and 10–20 cm soil. Root production and mortality showed clear seasonal patterns, both peaking in the late growing season, with root production reaching its maximum in August and mortality peaking in September. These seasonal dynamics were mainly associated with variations in growing-season precipitation and were not markedly altered by mowing intensity. However, root production and turnover rate were affected by mowing treatment and soil depth. In the 0–10 cm depth, light mowing produced the highest cumulative root production and root turnover rate, which increased by 20% and 18% compared with the no mowing, respectively. In contrast, the mowing effect was weaker in the 10–20 cm soil depth. Structural equation modeling indicated that mowing influenced root production through both direct and indirect pathways, with soil water content, soil temperature, and the fraction of belowground net primary production playing key roles. Overall, light mowing had a positive effect on fine root production and accelerated root turnover without disrupting seasonal root dynamics, indicating its potential to optimize belowground carbon allocation and sustain root-mediated soil carbon inputs in saline-alkali grasslands. These findings provide new evidence for identifying sustainable mowing thresholds in fragile grassland ecosystems.

## 1 Introduction

Mowing is one of the most common management practices in grasslands, yet its effects on fine-root dynamics remain poorly understood. Fine roots are a major source of soil carbon input because their production, mortality, and turnover directly contribute to belowground carbon cycling, especially in temperate grasslands where substantial photosynthate is allocated to root systems (Bai et al., 2015; Dijkstra et al., 2021; Gherardi and Sala, 2020; Pausch and Kuzyakov, 2018; Sokol et al., 2019). Fine roots also play key roles in nutrient uptake, water acquisition, and plant-soil interactions (Cordeiro et al., 2026; Freschet et al., 2021; Terrer et al., 2021). However, because belowground processes are difficult to observe directly, it remains unclear how mowing influences fine-root production, mortality, turnover, and seasonal dynamics. Addressing this knowledge gap is essential for understanding the ecological consequences of mowing and for guiding sustainable management of saline-alkali grasslands.

The intensity of mowing is a decisive factor governing the balance between aboveground biomass recovery and belowground carbon sequestration (Conant et al., 2017; Ward et al., 2016). Saline-alkali grasslands account for a large proportion of global marginal lands. Managing the intensity of such disturbances is especially crucial in these ecosystems, as they feature harsh soil physicochemical conditions including high pH levels and salt-triggered osmotic stress (Litalien and Zeeb, 2020). Root systems in these ecosystems are already forced to allocate substantial metabolic energy to osmotic adjustment and ion exclusion to survive salt toxicity (Munns and Tester, 2008; Van Zelm et al., 2020). Consequently, intensive mowing may impose a “double stress” by depriving plants of their photosynthetic tissues, thereby depleting the non-structural carbohydrate reserves required for root maintenance and stress tolerance (Martínez-Vilalta et al., 2016). While the “compensatory growth hypothesis” suggests that moderate levels of disturbance can stimulate root proliferation through shifted biomass allocation (Ferraro and Oesterheld, 2002; McNaughton, 1983), empirical evidence remains highly inconsistent. Chronic mowing has been shown to significantly decrease root biomass and alter root turnover (Wan and Luo, 2003). However, these effects are highly context-dependent, with the magnitude and direction of the response mediated by local precipitation, soil fertility, and overall site production (Gill and Jackson, 2000). Notably, most existing research has focused on non-saline grasslands (Luo et al., 2021; Wei et al., 2019), which fail to capture the complex, long-term *in situ* dynamics of fine roots under the fluctuating conditions of saline-alkali soils.

The mechanisms by which mowing regulates fine root production and turnover are multifaceted, involving both biotic and abiotic pathways (Gill and Jackson, 2000; Wang et al., 2019). On the biotic side, mowing significantly alters the allocation patterns of photosynthates (Luo et al., 2021). According to the “optimal partitioning theory,” plants may increase the fraction of belowground net primary production (*f*_BNPP_) to compensate for resource limitation, yet excessive biomass removal can deplete non-structural carbohydrate reserves, eventually suppressing root regrowth (Poorter et al., 2012). Beyond individual physiological responses, mowing intensity also dictates root dynamics by shifting plant community composition. For instance, a decrease in the ratio of grasses to forbs can significantly alter ecosystem-level root turnover, as these functional groups possess distinct root morphological traits and life-history strategies (Bardgett et al., 2014; Tjoelker et al., 2005). On the abiotic side, mowing modifies the microclimate and soil physicochemical environment. The removal of canopy cover increases soil temperature and evaporative demand, which, in saline-alkali grasslands, can exacerbate salt accumulation in the topsoil through capillary action (Lin et al., 2016). Such shifts in soil moisture, temperature, and salinity levels act as critical regulators of root phenology and lifespan (Eissenstat et al., 2013). Despite the importance of these pathways, how biotic and abiotic factors interact to determine the threshold of mowing intensity in salt-stressed ecosystems remains a significant knowledge gap.

To address these knowledge gaps, we conducted a two-year field experiment in a saline-alkali grassland of northern China. By implementing a gradient of mowing intensities, we aimed to determine the optimal management threshold for maintaining root production and turnover. We utilized the improved windows method for high-frequency, *in situ* monitoring of fine root production and turnover, providing a precise temporal perspective of root dynamics. Our specific objectives were: (1) to quantify the responses of root seasonal dynamic patterns, fine root production and turnover rates to mowing intensities; and (2) to identify how biotic factors (e.g., *f*_BNPP_ and plant community composition) and abiotic soil properties (e.g., soil water and soil temperature) collectively influence these processes. We hypothesized that: (1) light mowing would promote fine root production and root turnover, and maintain relatively stable seasonal dynamics compared with no mowing and heavy mowing; and (2) the responses of fine root dynamics to mowing would be jointly mediated by shifts in biotic factors and abiotic soil conditions. This approach is expected to sustain the belowground carbon input necessary for ecosystem resilience without further compromising the stability of saline-alkali grasslands.

## 2 Materials and methods

### 2.1 Study area

This study was carried out at the Youyu Loess Plateau Grassland Ecosystem National Research Station (39°59′ N, 112°19′ E; elevation 1348 m), situated in Youyu County, Shanxi Province, Northern China. The region’s historical climate is characterized by a mean annual temperature of 4.6 °C and an average annual precipitation of 425 mm, the majority of which occurs during July and August. Although thermal conditions remained stable throughout the two-year study, however, there were significant differences in precipitation between years: 2018 was a relatively wet year (490 mm), while 2019 was substantially drier, receiving only 310 mm (**Supplementary Figure 1**). The local vegetation is dominated by a perennial rhizomatous grass, *Leymus secalinus* (Georgi) Tzvel., which accounts for approximately 60% of the total aboveground productivity due to its superior resilience to drought and salinity (Diao et al., 2025a). Other common constituents include the highly salt-tolerant but drought-sensitive tufted grass *Puccinellia tenuiflora* (Griseb.) Scribn. & Merr., along with various forb assemblages. Classified as chestnut soil under the Chinese Soil Taxonomy, the substrate is highly alkaline (pH 9.0; EC 3.4 ms cm^−1^) (Diao et al., 2025b). Initial topsoil analysis (0–10 cm) revealed concentrations of total nitrogen, total phosphorus, and organic carbon at 0.8 g kg^−1^, 0.39 g kg^−1^, and 5.43 g kg^−1^, respectively (Diao et al., 2025a).

### 2.2 Experimental design

A randomized complete block design was employed in the present study, with four mowing intensity levels set as follows: no mowing (M0), light mowing (M1, stubble height of 10 cm), moderate mowing (M2, stubble height of 5 cm), and heavy mowing (M3, stubble height of 2 cm). Four replications were assigned to each treatment, giving a total of 16 experimental plots that were uniformly rectangular and measured 2 m × 12 m (24 m^2^). The spacing between plots was 2 meters. Mowing was conducted in mid-to-late August each year, consistent with the local hay-making schedule for agricultural and animal husbandry production.

### 2.3 Sample collection

#### 2.3.1 Measurements of root dynamics

To evaluate root dynamics, we adopted the modified root-window method as described by Bai et al. (2008, 2015) and Cui et al. (2024). In September 2017, vertical glass windows (dimensions: 28 cm × 20 cm × 0.3 cm) were installed in the experimental plots post-growing season. Each pane featured two 10 × 10 cm quadrats marked by 2 mm etched grooves, situated 8 cm above the bottom, 2 cm below the top, and 5 cm from both the left and right sides. To minimize external artifacts, black iron sheets (20 cm × 2 cm) were utilized to seal the gaps at the soil-glass boundary, thereby protecting the root systems from light interference and moisture seepage.

Within each plot, a vertical pit was excavated to accommodate the glass window. The pane was pressed firmly against the soil face and held in place with iron stakes. We then backfilled the pit and compacted the soil to restore its original bulk density. These windows remained undisturbed throughout the study period. To capture root images, we used an HP Scanjet G2410 scanner (Hewlett-Packard, Palo Alto, CA, USA) positioned 1 cm from the glass. Monitoring began on May 2, 2018, approximately eight months after installation, and continued every 15 days until October 19, 2019. At each sampling interval, the backfill was carefully removed and the glass surface was wiped clean with a soft cloth. Once scanning was complete, the soil was replaced and compacted using the same method as before.

Root dynamics, including appearance and senescence, were quantified using MapInfo Professional 7.0 (Pitney Bowes MapInfo Corporation, New York, USA). Each individual root identified in the baseline images was assigned a unique identification code. By comparing images from subsequent sampling intervals with the initial reference scans, newly emerged roots were distinguished from the existing population and labeled with new codes. Roots that were no longer visible in later scans were classified as dead or decomposed. Root production for each interval was defined as the total length of all new primary roots and lateral branches. Mortality was determined by measuring the combined length of roots that senesced or vanished during the observation period. Standing crop was estimated by calculating the difference between cumulative production and cumulative mortality throughout the growing season. Finally, root longevity was recorded as the number of days between the initial appearance of a root and its final disappearance from the images.

#### 2.3.2 Soil moisture and soil temperature

Soil water content (V, %) within the top 0–10 cm layer was determined using a portable TDR100 time-domain reflectometer (Campbell Scientific, Germany) fitted with a 7.5 cm probe. Data collection followed a 15-day cycle, synchronized with the root scanning schedule. Within each plot, five replicates were recorded in the vicinity of the root window. The mean of these five readings was then calculated and used for all subsequent statistical analyses.

### 2.4 Data analysis

We tested the normality and homoscedasticity of the dataset with the Shapiro-Wilk test prior to further analysis. Two-way ANOVA was employed to evaluate how mowing intensity, soil layer, and their interaction influence root production, mortality, standing crop, and turnover; where significant interactive effects were detected, one-way ANOVA was applied to quantify the independent contributions of each variable. Kaplan-Meier survival analysis was used to estimate mean root lifespan using the Oisurv package. To link root production and turnover to biotic and abiotic factors, Mantel’s r statistic and Spearman’s correlations were analyzed using the “linkET”, ‘Vegan”, and “dplyr” R packages. To further discern the direct and indirect effects the biotic and abiotic factors drivers on root production and turnover structural equation modeling (SEM) was performed. In brief, we examined the impact paths of mowing treatments on biotic and abiotic factors, followed by root production and turnover. We first considered a hypothetical conceptual model that included all reasonable paths. Then, we sequentially eliminated unimportant paths in order, unless these paths were biologically meaningful; alternatively, we added paths based on residual correlations. This process was repeated until the model fully matched the data, with the p values >0.05 (that is, there was no significant discrepancy between the predicted model and the observed data) and a root mean square error of approximation (rmsea) < 0.05. The SEM-related analysis was performed using the “lavaan” R package. All statistical analyses were performed using R (Version 4.6.0, R Core Team).

## 3 Results

### 3.1 The seasonal dynamics on root production, mortality and standing crop

**Figure 1:**
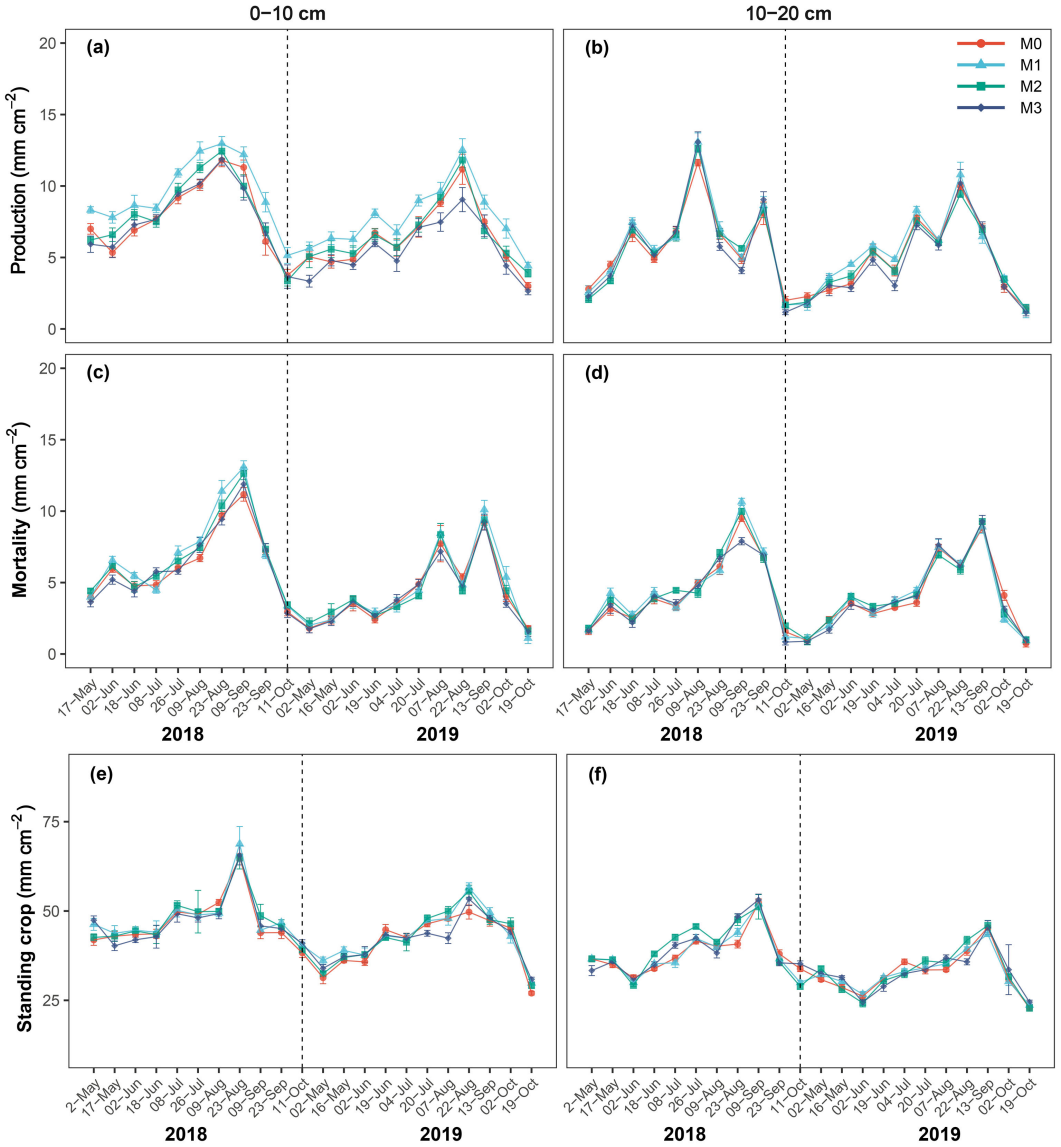
Seasonal dynamics of root production, root mortality, and average root standing stock under different grazing intensities and soil layers. The data are presented as mean ± SE for 0–10 cm and 10–20 cm soil depths during the mowing seasons from May 2, 2018, to October 19, 2019. M0, M1, M2, and M3 represent no mowing, light mowing, moderate mowing, and heavy mowing, respectively. The images **(a, c, e)** represent root production, mortality, and standing crop at a depth of 0-10 cm, while images **(b, d, f)** represent root production, mortality, and standing crop at a depth of 10-20 cm.

For mowing intensities, the root production and mortality of plant communities in northern saline-alkali grasslands demonstrated distinct seasonal dynamics, which were mainly influenced by precipitation variations. Specifically, during two consecutive years of *in-situ* monitoring, root production in both the 0–10 cm and 10–20 cm soil layers peaked in August and then gradually declined (**Figures 1a, b**). In contrast, root mortality reached its maximum in September, showing a delayed peak relative to root production (**Figures 1c, d**). Similarly, variations in mowing intensity did not significantly alter the overall seasonal patterns of root production and mortality. Root standing crop followed a similar trend to root production, peaking in August and then gradually declining (**Figures 1e, f**).

### 3.2 The root production, root mortality, and root standing crop

**Figure 2:**
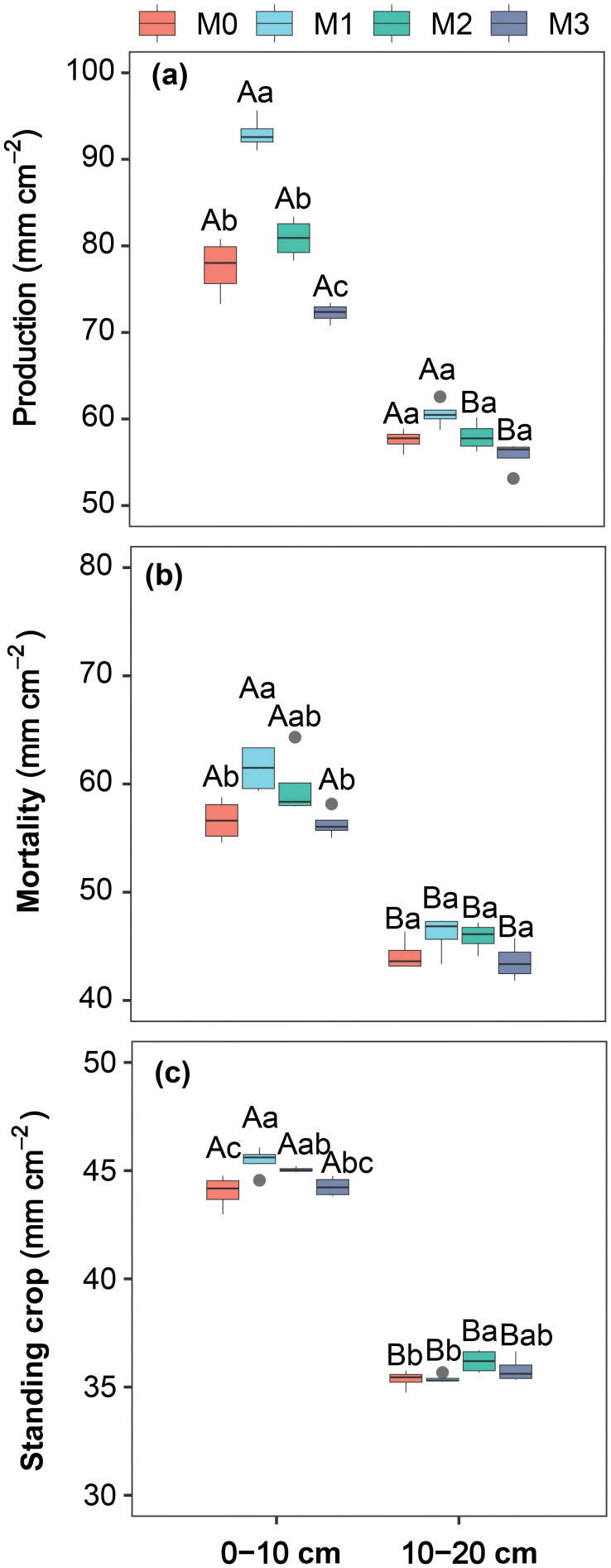
Cumulative root production **(a)**, cumulative root mortality **(b)**, mean root standing crop **(c)** under mowing intensities and soil depth during the growing seasons from May 2, 2018 to Oct. 19, 2019. Data are mean ± SE with n = 4. lowercase letters above the boxplots indicate significant differences among mowing intensities. Uppercase letters above the bar charts indicate significant differences among soil depths (*p* < 0.05). M0, M1, M2, M3 represent no mowing, light mowing, moderate mowing, heavy mowing.

During two consecutive years of continuous monitoring, cumulative root production and mortality ranged from 53.1 to 95.7 mm cm^-2^ and 41.8 to 64.3 mm cm^-2^, respectively. Cumulative root production was significantly affected by mowing treatment, soil layer, and their interaction (*p* < 0.05, **Supplementary Table 1**). In contrast, cumulative root mortality was only significantly influenced by mowing treatment and soil layer (*p* < 0.05), with no significant interaction effect between the two (*p*>0.05, **Supplementary Table 1**). In the 0–10 cm soil layer, cumulative root production was highest under light mowing, significantly higher than the other three mowing treatments by 20.0% (M0), 15.0% (M2), and 28.8% (M3). In the 10–20 cm soil layer, although no significant differences were observed among the four mowing treatments, light mowing still yielded the highest value (**Figure 2a**). For cumulative root mortality, the pattern was similar to that of cumulative root production: it was highest under light mowing in the 0–10 cm layer, significantly higher than M0 and M3 by 8.3% and 9.1%, respectively. No significant differences were detected among the four mowing treatments in the 10–20 cm layer (**Figure 2b**). Mean root standing crop under light mowing was significantly higher than M0 and M3 in the 0–10 cm layer by 3.4% and 2.8%. Notably, in the 10–20 cm layer, moderate mowing resulted in the highest root standing crop, significantly exceeding M0 and M1 by 2.4% and 2.3% (**Figure 2c**).

### 3.3 Annual root turnover and mean root lifespan

**Figure 3:**
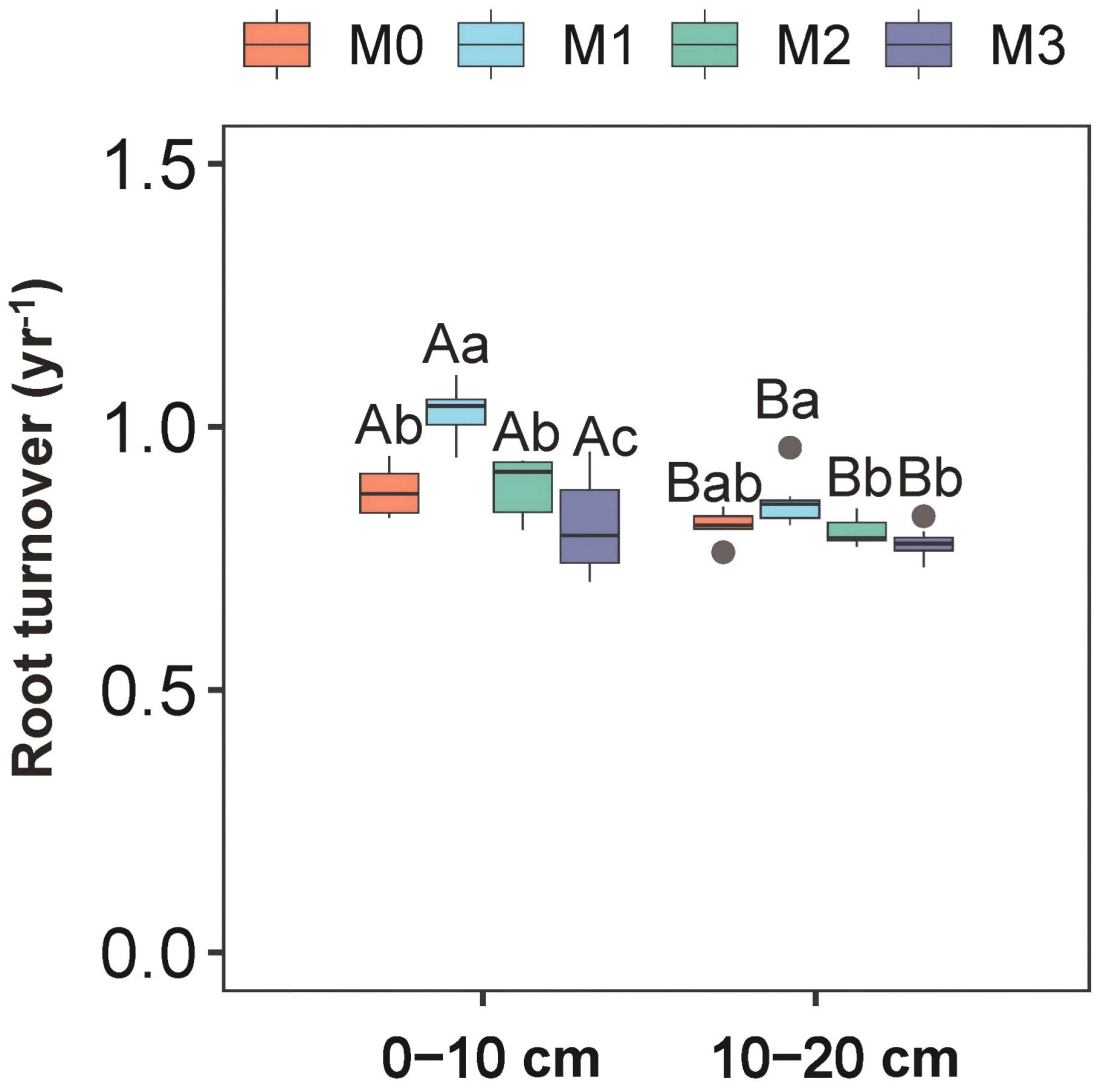
Annual root turnover (times yr^-1^, mean ± SE) of 0–20 cm at different mowing intensities **(a)** and different years **(b)**. Different lowercase letters above the boxplots indicate significant differences among different mowing intensities. Different uppercase letters above the bar charts indicate significant differences among different soil layers (*p* < 0.05). M0, M1, M2, M3 represent no mowing, light mowing, moderate mowing, heavy mowing.

For root turnover rate, during two consecutive years of field experiments, it ranged from 0.71 to 1.1 yr^-1^, and was significantly affected by mowing treatment, soil layer, and their interaction (*p* < 0.01, **Supplementary Table 1**). Root turnover rate was highest under light mowing in both soil layers. Specifically, in the 0–10 cm soil layer, light mowing was significantly higher than the other three treatments by 18.0% (M0), 15.7% (M2), and 27.2 (%). In the 10–20 cm soil layer, it was significantly higher than M2 and M3 by 7.5% and 10.3%, respectively (**Figure 3**).

**Figure 4:**
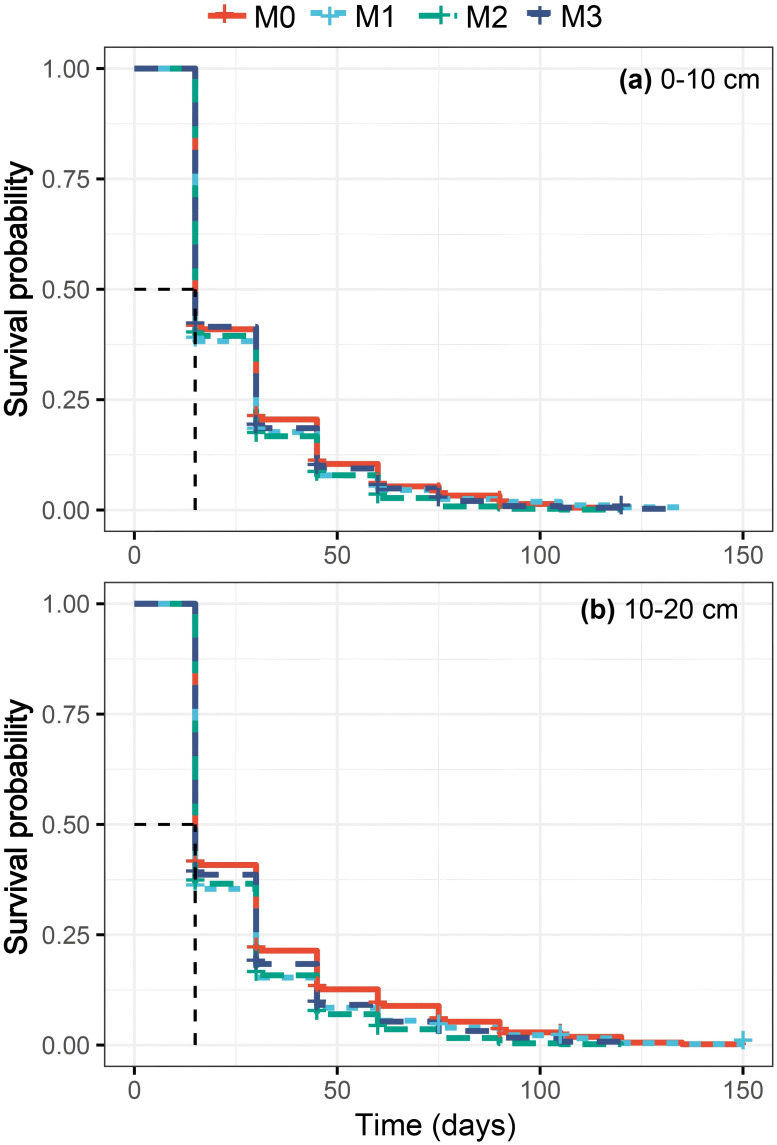
Survival curves of roots arising in the 0–10 cm **(a)** and 10–20 cm **(b)** soil depths under different mowing treatments. The mean and median lifespan were calculated via the survival curves using Kaplan-Meier analysis. The log-rank test was performed to compare the effects of different treatments on the root survival rate. M0, M1, M2, M3 represent no mowing, light mowing, moderate mowing, heavy mowing.

Mowing intensity had a significant effect on root lifespan in the 0–10 cm and 10–20 cm soil layers (*p* < 0.05). Under no mowing (G0), the mean root lifespan was the longest, at 12.36 days (0–10 cm) and 14.2 days (10–20 cm). More than 60% of roots survived for less than 20 days, and nearly all roots had died by day 90. Regarding root survival probability, M3 achieved the highest rate, while M1 had the lowest in 0–10 cm. It is worth noting that the maximum root lifespan was recorded under M2, reaching 135 days (**Figure 4a**). In contrast, in the 10–20 cm soil layer, M0 had the highest root survival rate, while the maximum root lifespan was recorded under M2, reaching 150 days (**Figure 4b**).

### 3.4 Pathways by which mowing intensities affects the response of BNPP and root turnover

**Figure 5:**
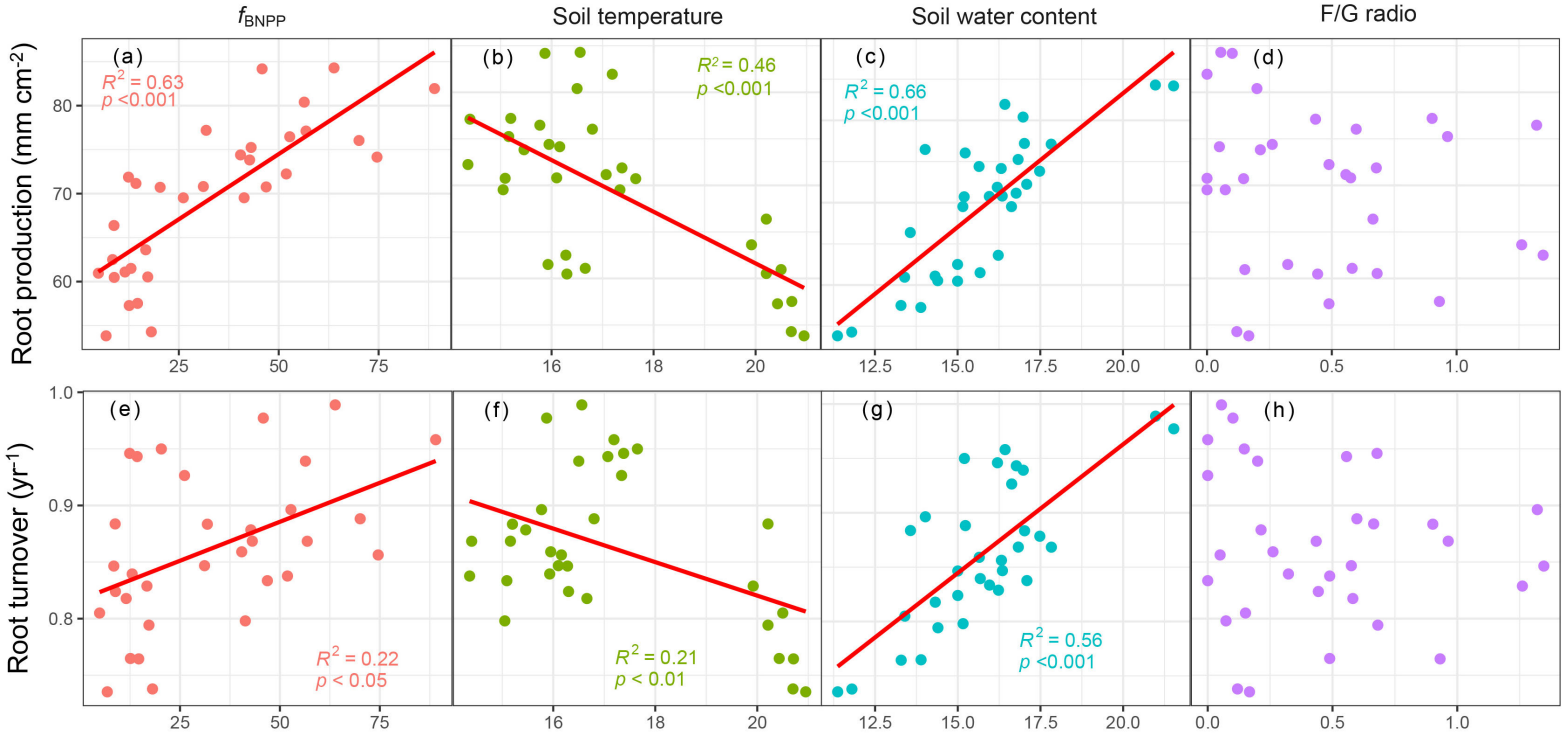
Regressions of root production and root turnover on *f*_BNPP_ **(a, e)**, soil temperature **(b, f)**, soil water content **(c, g)** and F/G ratio **(d, h)**. Data for root production were the cumulative values in the 0–20 cm soil layer from a two-year consecutive field experiment. The data of soil temperature, soil water content and F/G radio were those mean values in the experimental plots in the top soil depths during the growing seasons across two years.

**Figure 6:**
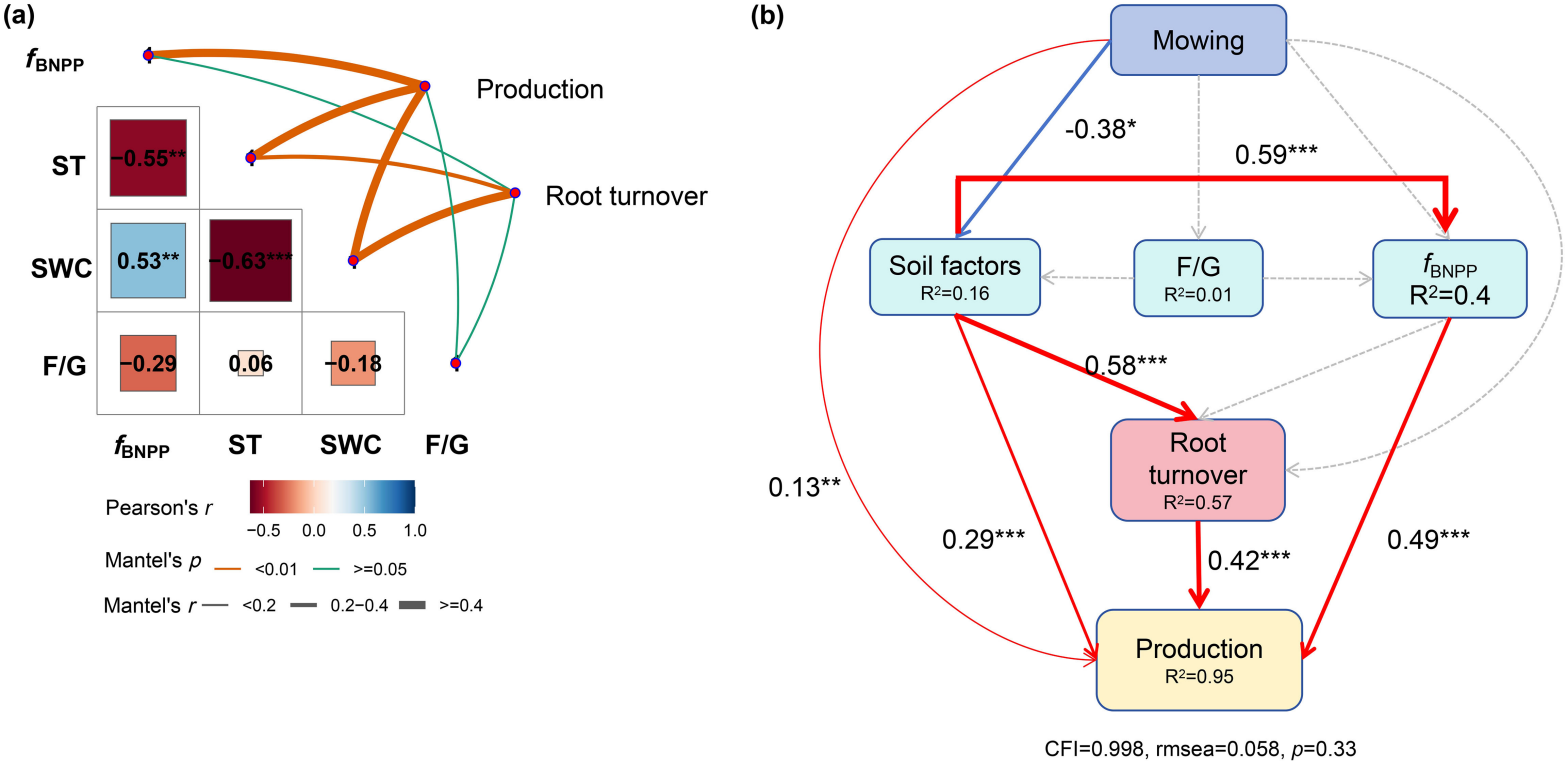
Environmental drivers of root production and root turnover in the mowing experiment. **(a)** Correlations between root production and root turnover and environmental variables. Edge width corresponds to the absolute value of the correlation coefficient determined by the linear mixed­effects models. The colors of each line and block indicate the direction and magnitude of the correlation. The colored and gray lines denote significant and nonsignificant correlations, respectively. Pairwise comparisons of environmental factors are shown in the triangular grid, with each color denoting the value of the Pearson’s correlation coefficient. **(b)** Structural equation modeling showing the effect pathways among mowing treatments, soil factor, F/G ratio, *f*_BNPP_, root production and root turnover. The red and blue arrows indicate positive and negative relationships, respectively. The solid and dashed lines indicate significant (*p* < 0.05) and nonsignificant (*p>*0.05) relationships. The numbers labeling the pathway arrows indicate the standard path coefficients. R^2^ represents the proportion of variance explained for every dependent variable. rmsea=0.058, *p* = 0.33 (larger p values indicate that the predicted model and observed data are equal, i.e., the model is a good fit), comparative fit index (CFI) =0.998. F/G ratio, biomass ratio of forb to grass; Soil factors, the scores of the first axis derived from principal component analysis (PCA) of soil temperature and soil moisture content. All variables except soil factors were standardized before the SEM analysis.

*f*_BNPP_ (*R^2^* = 0.63, *p* < 0.001) and soil water content (*R^2^* = 0.66, *p* < 0.001) were positively correlated with root production, whereas soil temperature exhibited a significant negative correlation (*R^2^* = 0.46, *p* < 0.001) (**Figures 5a–c**). Similar to root production, root turnover also showed positive correlations with *f*_BNPP_ (*R^2^* = 0.22, *p* < 0.05) and soil water content (*R^2^* = 0.56, *p* < 0.001), while soil temperature was negatively correlated with root turnover (*R^2^* = 0.21, *p* < 0.01) (**Figures 5e, f**). Notably, the F/G ratio showed no significant correlation with either root production or turnover (*p*>0.05; **Figures 5d, h**). To understand how mowing-induced changes in abiotic and biotic factors affect root production and root turnover, we conducted a Mante’s R statistic based on the data in this mowing gradient experiment. Among the examined factors, *f*_BNPP_, soil temperature, and soil water content significantly affected the response of root production to changes in mowing. For root turnover, only soil temperature and soil water content exerted significant effects. The F/G had no significant influence on either of them (**Figure 6a**). We further evaluated the pathways and the relative roles of these factors in affecting root production by a SEM analysis. The results demonstrated that mowing exerted a direct positive effect on root production. Meanwhile, increasing mowing intensity indirectly promoted root production by suppressing soil factors. Alternatively, the inhibition of soil factors could also boost root production through indirect pathways, namely by increasing *f*_BNPP_ or accelerating root turnover (**Figure 6b**). Similarly, the F/G ratio did not participate in the SEM, as it had no significant correlation with any of the factors. That is to say, the effect of mowing on root production was not significantly related to the structure and composition of the plant community-at least statistically speaking.

## 4 Discussion

In this study, we used the improved root window method to conduct *in-situ* monitoring of root dynamics, including root production, mortality, standing crop, and turnover, in order to evaluate the effects of different mowing treatments in the northern China saline-alkali grassland. Our results indicate that both root production and mortality exhibited significant seasonal dynamics over time, and these patterns were not significantly affected by varying mowing intensities (**Figures 1a–d**). Specifically, root production in two soil layers peaked in August and then gradually declined (**Figures 1a, b**). This was mainly attributed to the favorable precipitation and relatively high temperatures in summer, which stimulated rapid root growth to meet the water and nutrient demands of aboveground plant parts (**Supplementary Figure 2**; Bai et al., 2015; Cui et al., 2024). In addition, the occurrence of this peak is also strongly related to the dominance of perennial rhizomatous grasses (e.g., *Leymus secalinus*) in this region. After completing seed reproduction, this species tends to allocate more photosynthetic products to roots to ensure successful regreening in the following year. However, in contrast to previous studies, we did not observe an obvious root growth peak in spring (Bai et al., 2015; McCormack et al., 2014). In other words, the seasonal pattern observed in this study was unimodal rather than bimodal (Cui et al., 2024). This was largely because abundant rainfall in the spring of 2018 was sufficient to support aboveground plant growth, eliminating the need for plants to produce additional roots (**Supplementary Figure 1**). In 2019, despite lower spring precipitation, the legacy effect of rainfall from the previous year persisted. Together, these factors resulted in the absence of a secondary root production peak in spring (Sun et al., 2024). Similar to root production, root mortality also showed a clear unimodal pattern, generally peaking in September, slightly later than root production. Previous studies have indicated that these two processes often occur synchronously and exhibit a strong positive correlation, which results in their similar seasonal dynamic patterns (Kwatcho Kengdo et al., 2025; Luo et al., 2021; Primka et al., 2022). This is the key reason why root mortality showed only one peak. Furthermore, these results also indicate that the seasonal dynamics of roots were only strongly affected by variations in precipitation during the growing season, and showed no significant correlation with mowing treatments.

For mowing intensities, our results showed that light mowing significantly increased root production compared with the control treatment, while heavy mowing markedly reduced it. Such effects on root production were mainly concentrated in the 0–10 cm soil layer and were not significant in the 10–20 cm soil layer (**Figure 2a**). Several potential mechanisms may account for this phenomenon. First, the structural equation modeling (SEM) results revealed that mowing intensity did not affect root production via the F/G ratio (**Figure 6b**, **Supplementary Figure 4**). In other words, mowing could not regulate root production in a short term by altering plant community composition. Meanwhile, this result indicated that increasing mowing intensity exerted a direct positive effect on root production. We attributed this pattern to the differences in plant compensatory growth induced by varied mowing intensities. Previous studies have demonstrated that mowing triggers compensatory growth by removing aboveground photosynthetic tissues (Hawkes and Sullivan, 2001; Maschinski and Whitham, 1989; Sun et al., 2021). The rapid recovery of aboveground biomass requires substantial water and nutrient supply from roots, thereby promoting the increase of root production. However, this compensatory growth effect gradually weakens as mowing intensity rises. As the dominant species in this region, *Leymus secalinus* is a perennial rhizomatous grass. Severe loss of aboveground photosynthetic organs cannot support the rapid recovery of plants within a short period. In addition, heavy mowing may reduce the availability of stored carbon reserves for recovery, although non-structural carbohydrates were not measured in this study and this should therefore be considered a possible mechanism rather than a confirmed explanation. Moreover, mowing may also affect root production indirectly by altering soil physicochemical properties (Luo et al., 2021). This study found that the impact of mowing on root production mainly occurs through the following two pathways. First, the direct pathway: with the increase of mowing intensity, the removal of aboveground photosynthetic organs leads to increased surface exposure, which in turn causes an increase in soil temperature and a decrease in soil moisture content (**Figure 6**; **Supplementary Figure 2**). Our results showed a significant negative correlation between root production and soil temperature, and a positive correlation with soil moisture content (**Figure 5**), which is consistent with previous studies that increased temperature and drought stress inhibit root growth (Bai et al., 2010; de Vries et al., 2016; Frank, 2007; Zhou et al., 2011). Second, the indirect pathway: changes in soil physicochemical factors mediated by mowing inevitably lead to alterations in soil resources, which synchronously disrupt the original balance of aboveground and underground resources. The functional balance model suggests that plants allocate more photosynthates to aboveground parts when light is sufficient, and to roots when soil resources are abundant (Chapin et al., 1987; Reynolds and Thornley, 1982; Tilman, 1988);. The results of SEM indicated that, across the full mowing-intensity gradient, increasing mowing intensity had an overall negative effect on soil conditions, leading to a relative reduction in belowground resource availability (**Figure 6**). Correspondingly, the relative increase in aboveground resource allocation may have promoted the transfer of photosynthates to aboveground tissues, thereby reducing *f*_BNPP_ (**Supplementary Figure 3**). However, this overall negative pathway does not preclude treatment-specific differences among mowing levels. In particular, light mowing appeared to maintain relatively more favorable soil temperature and moisture conditions than moderate and heavy mowing, which may have contributed to the significant increase in BNPP under light mowing (**Supplementary Figure 2**). Therefore, the effect of mowing on soil factors should be interpreted as intensity-dependent and non-linear: light mowing may preserve a relatively suitable soil microenvironment, whereas stronger mowing intensities tend to suppress soil conditions overall.

Root turnover generally refers to the dynamic processes of root growth, mortality and decomposition, and it is usually inversely proportional to root lifespan (Eissenstat et al., 2013). In general, root turnover inputs carbon and nutrients into soil and serves as one of the major processes of carbon sequestration in terrestrial ecosystems (Tefs and Gleixner, 2012; Jackson et al., 2017; Richter et al., 1999). In particular, fine roots (diameter < 2 mm) have a rapid turnover rate and are recognized to play a crucial role in carbon and nutrient cycling of terrestrial ecosystems (Richter et al., 1999). Current research on root turnover in grassland ecosystems is still limited, especially studies concerning the responses of root turnover to different mowing intensities. This undoubtedly greatly hinders our understanding of belowground processes in grassland ecosystems. Meanwhile, root monitoring faces considerable practical challenges; for example, inconsistent methodologies among existing studies lead to large discrepancies in research results (Bai et al., 2015; Cui et al., 2024; Gao et al., 2008). In this study, we adopted *in situ* root measurement technology to continuously monitor root growth dynamics over time, aiming to evaluate the effects of different mowing intensities on root turnover and root lifespan in saline-alkali grasslands of northern China. The results showed that light mowing increased root turnover, which was mainly achieved by altering soil physicochemical properties. Specifically, light mowing removed aboveground plant tissues, thereby increasing soil temperature and reducing soil moisture content (**Figure 6a**; **Supplementary Figure 2**). According to the functional balance model, the reduction in belowground resources drives plants to allocate more photosynthates to the aboveground part with relatively abundant resources (Chapin et al., 1987; Tilman, 1988). This reduces carbon allocation to roots and consequently accelerates root turnover (**Figure 4**). On the other hand, the loss of aboveground photosynthetic tissues forces plants to prioritize the allocation of photosynthates to aboveground growth. Based on the cost-benefit theory, plants tend to minimize carbon consumption on roots to maximize overall growth (Eissenstat and Yanai, 1997). This accelerates root turnover because the carbon cost of maintaining old roots far exceeds that of producing new roots (Yanai et al., 1995). Additionally, rapid root turnover also promotes the accumulation of soil nutrients (Dijkstra et al., 2021; Gill and Jackson, 2000). However, with the increase in mowing intensity, aboveground photosynthetic tissues are almost completely removed and cannot recover rapidly in a short period, which greatly constrains plant photosynthesis. Under such conditions, plants tend to retain old roots and reduce the production of new roots to conserve photosynthate reserves, which is essential for plant normal growth in the subsequent year (Luo et al., 2021). For different soil layers, our results showed that root lifespan in the 10–20 cm soil layer was longer than that in the 0–10 cm soil layer (**Figure 4**). This is mainly because roots in the surface layer contribute more to water and nutrient absorption (Hodge et al., 2000; Steudle, 2000), that is, they are mainly composed of fine roots (Jackson et al., 1997). Secondly, to ensure water and nutrient absorption, a large number of annual plants tend to accumulate their roots in the 0–10 cm soil layer (Padilla and Pugnaire, 2007; Schenk and Jackson, 2002). This is because the extension of roots into the deep soil requires a large allocation of photosynthates, which is often unfavorable for annual plants (Bloom et al., 1985; Eissenstat and Yanai, 1997). Meanwhile, this soil layer is also highly susceptible to changes in soil environments (e.g., soil temperature and soil water content), which is another potential important factor (Schwinning and Ehleringer, 2001). Furthermore, our results showed that root survival probability under light mowing was the longest in 0–10 cm (**Figure 4a**). A possible explanation for this phenomenon is that the absolutely dominant species in this region (e.g., *Leymus secalinus*) were less affected by light mowing, and their aboveground photosynthetic organs recovered quickly after mowing. This allowed them to maintain an advantage in light competition, and as perennial plants, *Leymus secalinus* could still retain a higher root survival probability. Moreover, salt stress may influences soil organic carbon (SOC) dynamics by regulating root turnover. On the one hand, salinity-induced changes in fine-root turnover and rhizosphere carbon inputs can modify the replenishment of SOC (Kumar et al., 2023; Soda et al., 2017). On the other hand, salt stress also regulates microbial decomposition and the formation of microbially derived organic carbon, thereby influencing the efficiency with which newly added carbon is converted into stable SOC pools (Chari and Taylor, 2022; Jia et al., 2024; Kumar et al., 2023). Therefore, SOC dynamics under salt stress are not determined solely by the quantity of root-derived carbon inputs, but by the coupled interactions among root turnover, microbial metabolism, and carbon stabilization mechanisms.

## 5 Conclusion

This study demonstrated that fine root dynamics in saline-alkali grasslands exhibit pronounced seasonal patterns, with root production peaking in August and root mortality peaking in September, and these temporal trends were governed primarily by variations in growing-season precipitation rather than mowing intensity. Although mowing did not substantially alter the seasonal pattern of root dynamics, it significantly affected cumulative root production, mortality, standing crop, and turnover, particularly in the 0–10 cm soil layer. The results further showed that the response of root dynamics to mowing was jointly regulated by abiotic and biotic pathways. Soil water content, soil temperature, and belowground carbon allocation were the main factors associated with changes in root production and turnover, whereas shifts in community composition appeared to play a limited role. These findings suggest that light mowing may help maintain the balance between aboveground utilization and belowground carbon input, thereby supporting ecosystem functioning and resilience in saline-alkali grasslands. Overall, under the specific conditions of this two-year study at a single saline-alkaline grassland site, light mowing appeared to be more favorable than moderate or heavy mowing for maintaining fine root activity. However, this inference should be interpreted cautiously, as its applicability to other saline-alkaline grasslands with different species compositions, salinity levels, or precipitation regimes remains to be tested.

## Data Availability

The original contributions presented in the study are included in the article/**Supplementary Material**. Further inquiries can be directed to the corresponding author.
