# Supplementary material for: Light mowing supports fine-root production in a temperate saline-alkaline grassland soil
Source: Front Plant Sci. 2026 Jul 15;17:1892574. doi: 10.3389/fpls.2026.1892574 (PMC13414826; doi:10.3389/fpls.2026.1892574)
Supplement: Supplementary file 1 [file SupplementaryFile1.docx]

Supplementary Material

# Supplementary Figures and Tables

## Supplementary Figures

**Supplemental Fig. S1.** The annual climate variability of the region from 2018 to 2019. Values on each panel are total annual precipitation and mean temperature.

**Supplemental Fig. S2.** Soil water content (a, b), and soil temperature (c, d) under different mowing intensities from 2018 to 2019. Different capital letters indicate significant differences among years; different lowercase letters indicate significant differences among mowing treatments. M0, M1, M2, M3 represent no mowing, light mowing, moderate mowing, heavy mowing.

**Supplemental Fig. S3.** The ratio of belowground net primary productivity (BNPP) to net primary productivity (*f*_BNPP_) (a, b) under different mowing intensities from 2018 to 2019. Different capital letters indicate significant differences among years; different lowercase letters indicate significant differences among mowing treatments. M0, M1, M2, M3 represent no mowing, light mowing, moderate mowing, heavy mowing.

**Supplemental Fig. S4.** Biomass ratio of forb to grass responses to different mowing intensities from 2018 to 2019. Different capital letters indicate significant differences among years; different lowercase letters indicate significant differences among mowing treatments. M0, M1, M2, M3 represent no mowing, light mowing, moderate mowing, heavy mowing.


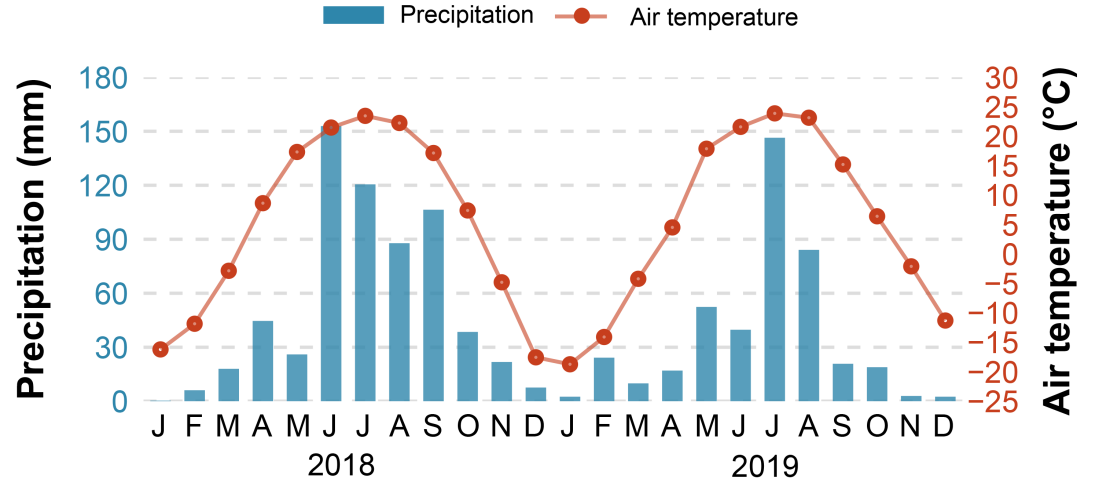


**Supplemental Fig. S1**


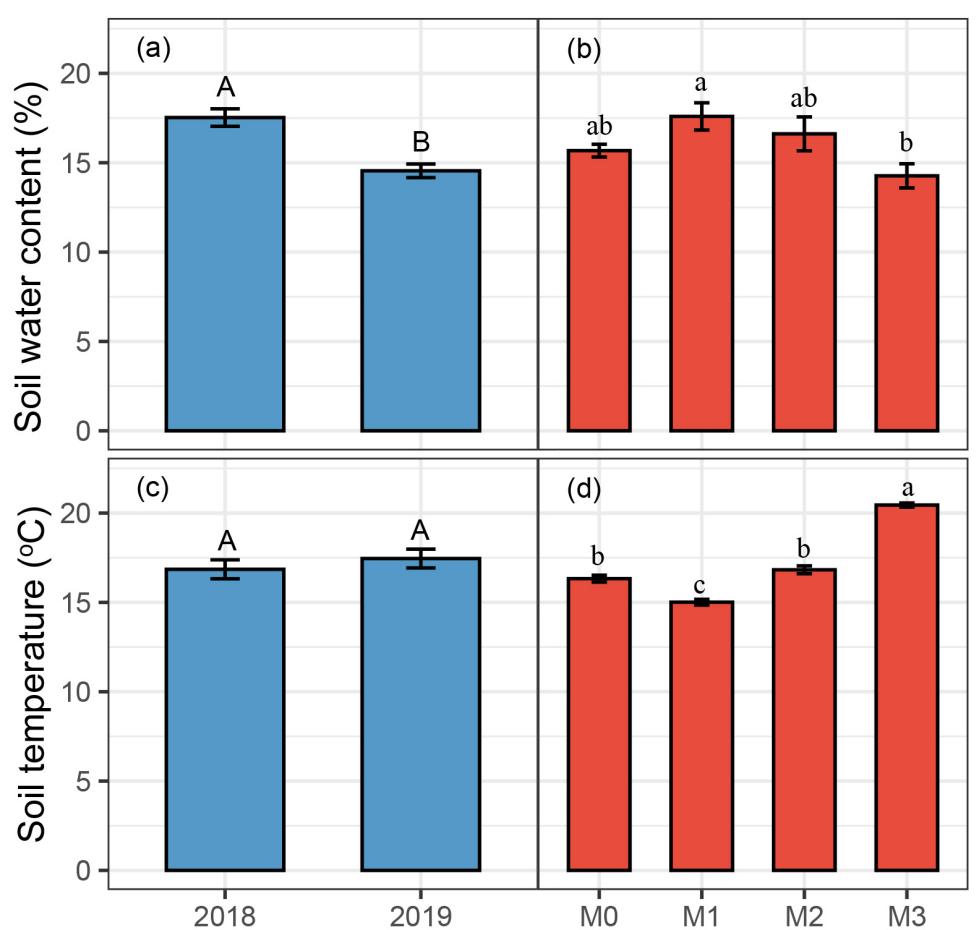


**Supplemental Fig. S2**


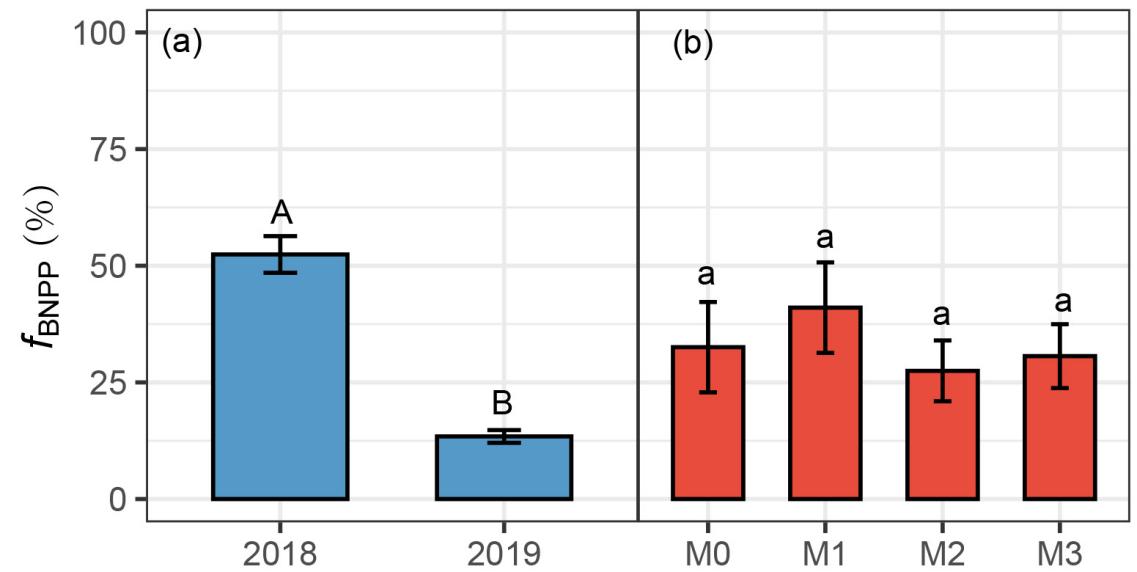


**Supplemental Fig. S3**


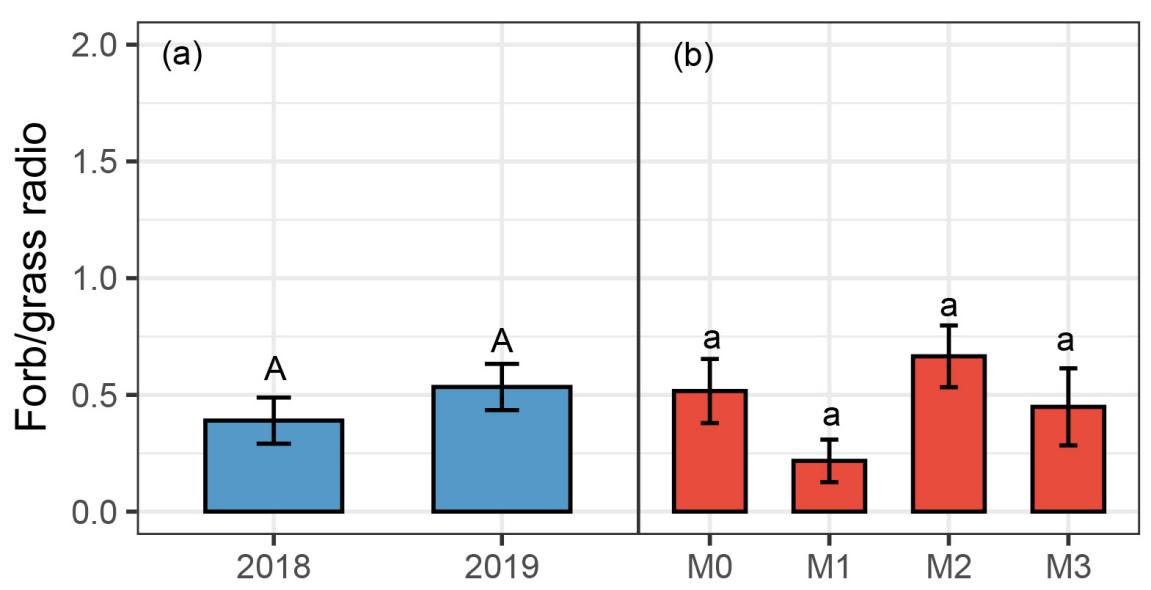


**Supplemental Fig. S4**

## Supplementary Table

**Table S1:** Results of two-ANOVA for effects of mowing intensities (M), soil depths (S) and their interactions on cumulative root production, cumulative root mortality, mean root standing crop, and root turnover.

|  |  | Root production | | Root mortality | | Root standing crop | | Root turnover | |
| --- | --- | --- | --- | --- | --- | --- | --- | --- | --- |
|  | df | *F* | Sig. | *F* | Sig. | *F* | Sig. | *F* | Sig. |
| G | 3 | 57.41 | <0.001 | 7.08 | <0.01 | 5.12 | <0.01 | 23.66 | <0.001 |
| S | 1 | 1042.4 | <0.001 | 385.11 | <0.001 | 2432.97 | <0.001 | 48.27 | <0.001 |
| G*S | 3 | 23.11 | <0.001 | 0.88 | 0.47ns | 3.98 | <0.05 | 5.39 | <0.01 |
